# Machine learning-based equations for improved body composition estimation in Indian adults

**DOI:** 10.1371/journal.pdig.0000671

**Published:** 2025-06-23

**Authors:** Nick Birk, Bharati Kulkarni, Santhi Bhogadi, Aastha Aggarwal, Gagandeep Kaur Walia, Vipin Gupta, Usha Rani, Hemant Mahajan, Sanjay Kinra, Poppy A. C. Mallinson

**Affiliations:** 1 Department of Non-Communicable Disease Epidemiology, London School of Hygiene & Tropical Medicine, London, United Kingdom; 2 Reproductive and Child Health and Nutrition, Indian Council of Medical Research, New Delhi, India; 3 Indian Institute of Public Health Bengaluru, Bengaluru, India; 4 Public Health Foundation of India, New Delhi, India; 5 Department of Anthropology, University of Delhi, New Delhi, India; 6 Clinical Division, ICMR National Institute of Nutrition, Hyderabad, India; University of Pittsburgh Department of Surgery, UNITED STATES OF AMERICA

## Abstract

Bioelectrical impedance analysis (BIA) is commonly used as a lower-cost measurement of body composition as compared to dual-energy X-ray absorptiometry (DXA) in large-scale epidemiological studies. However, existing equations for body composition based on BIA measures may not generalize well to all populations. We combined BIA measurements (TANITA BC-418) with skinfold thickness, body circumferences, and grip strength to develop equations to predict six DXA-measured body composition parameters in a cohort of Indian adults using machine learning techniques. The participants were split into training (80%, 1297 males and 1133 females) and testing (20%, 318 males and 289 females) data to develop and validate the performance of equations for total body fat mass (kg), total body lean mass (kg), total body fat percentage (%), trunk fat percentage (%), L1-L4 fat percentage (%), and total appendicular lean mass (kg), separately for males and females. Our novel equations outperformed existing equations for each of these body composition parameters. For example, the mean absolute error for total body fat mass was 1.808 kg for males and 2.054 kg for females using the TANITA’s built-in estimation algorithm, 2.105 kg for males and 2.995 kg for females using Durnin-Womersley equations, and 0.935 kg for males and 0.976 kg for females using our novel equations. Our findings demonstrate that supplementing body composition estimates from BIA devices with simple anthropometric measures can greatly improve the validity of BIA-measured body composition in South Asians. This approach could be extended to other BIA devices and populations to improve the performance of BIA devices. Our equations are made available for use by other researchers.

## Author summary

Bioelectrical Impedance Analysis, or BIA, is a popular technology for estimating the amount of fat and lean mass in the body. BIA scales are low-cost and portable, meaning they are commonly used in clinical weight monitoring and in large-scale research studies in place of more accurate but more expensive whole-body DXA scans. However, BIA technology was developed mostly in Western populations and relies on assumptions about body shape, size and composition that may not apply to all populations globally. We investigated whether addition of simple body measurements such as waist circumference and skinfold thickness could improve the accuracy of BIA measurements in an Indian population. We applied machine learning methods to a large dataset from India with simple body measurements and whole-body DXA scans to find the most informative combinations of measures. Our resulting models were able to estimate body fat and lean mass in Indians over twice as accurately as BIA scales alone, suggesting that this could be a valuable approach to improve body composition estimation from other BIA devices and in other under-represented populations. Our equations are available via a web application for researchers to use.

## Background

Measurements of body composition, such as body fat percentage, can provide meaningful health-related insights. Total body fat and intra-abdominal fat have been previously associated with metabolic syndrome [1], while lean body mass can be protective against chronic diseases and age-related adverse conditions [2,3]. Further, there is some evidence that these relationships are particularly pronounced in adults of Indian and South Asian ethnicities [1,2], underscoring the importance of accurately recording these body composition metrics in large-scale, international epidemiological studies. We note that the construct of ‘ethnicity’ is used in this manuscript to refer to people who have a shared ancestral region of origin, and may share certain traditions, diets, and values; we do not presume that any such differences are genetic in nature, or that broad ethnic groupings referred to here (e.g., Indian or European) represent a single ethnic or cultural group.

Bioelectrical impedance analysis (BIA) is commonly used as a feasible, lower-cost measurement of body composition in population-based studies as compared to dual-energy X-ray absorptiometry (DXA) and magnetic resonance imaging (MRI) [4]. Commercially and clinically available BIA devices, such as TANITA body composition analyzers, utilize inbuilt equations to estimate total and segmental lean and fat mass from a combination of body segment impedance values, sex, height, and weight [5]. However, these prediction equations were generally developed and validated in predominately European ethnic populations and may not generalize to other ethnic groups [6,7]. Generalizability to Asian Indians is a particular concern, because of a body composition characterized by higher abdominal and visceral adiposity and lower lean mass in this population [8–11]. A study of 200 healthy Asian Indian individuals found that in-built equations based on white ethnic populations from a TANITA analyzer underestimated total body fat percentage by 8.3%, or by 5.4% when using equations fit based on individuals of Japanese ethnicity, compared with DXA-derived values [12]. A study of 39 Indian adults found that BIA methods underestimated fat mass as compared to four-compartment model, suggesting population-specific equations are needed [13]. Another study of healthy Indian adult men found that existing BIA equations tended to underestimate body fat compared with deuterium oxide dilution as a reference method [14], while studies among Indian children have reported both under- and over- estimation of body fat percentage as compared to DXA-derived values [15–17].

This suggests a need for better methods to estimate body composition in Asian Indian populations [18]. We are aware of one study that developed a novel BIA-based equation to predict DXA-derived body fat among young adult Indian males [19], and another that developed lean body mass equations in people of Indian Asian ethnicity living in New Zealand [18], but both had relatively small samples. Furthermore, no Indian-specific equations are available for estimation of central body fat, which may be particularly relevant for understanding cardiometabolic risks in Indian adults. Although BIA is now the predominant low-cost method for measuring body composition in research and clinical settings, other popular approaches include equations based on anthropometric measures such as the Durnin-Womersley equations which use skinfold measurements [20]. Although there is evidence that these equations perform less well in Asian (and other non-white) ethnic groups compared with white populations [21], this indicates that using anthropometric measures in addition to BIA could potentially improve body composition estimation through recalibration of BIA estimates to specific populations [22].

We aimed to develop novel algorithms to estimate DXA-derived fat and lean mass for an adult Indian population, and in doing so establish whether simple anthropometric measures can improve the accuracy of BIA in an under-represented ethnic group. We conducted a cross-sectional study of 3037 adults in a community setting in India, who underwent assessment by whole-body DXA scan (the reference) alongside BIA (using TANITA BC-418) and various anthropometric measurements. We used the BIA-based default predictions as a performance baseline, and then incorporated additional predictors from the TANITA output alongside anthropometric measures related to body stature, adiposity, and muscularity, using machine learning approaches to explore non-linearities and identify the most parsimonious model, finally evaluating the accuracy of each against DXA-derived measures.

## Methods

### Study population

The present analysis makes use of data from the third wave of follow-up in the Andhra Pradesh Children and Parents Study (APCAPS), 2010–12. Details of the APCAPS have been published previously [23], but in brief the third wave sample includes all living children born in a defined area of Ranga Reddy district in 1987–90, and their siblings and parents, who agreed to take part in the study, and of whom a random subset were invited for DXA scanning in addition to undergoing anthropometric measurements and BIA. The APCAPS third wave follow-up study was conducted according to the guidelines laid down in the Declaration of Helsinki and all procedures involving human participants were approved by ethics committees of the Indian Council of Medical Research - National Institute of Nutrition, India (reference number: A2-2009), the Public Health Foundation of India, India (reference number 52/10), and the London School of Hygiene and Tropical Medicine, UK (reference number: 6471). Written informed consent was obtained from all participants (or witnessed thumbprint if illiterate).

### Data collection

A portable TANITA bioelectrical impedance analyzer (model BC-418 M57NA, TANITA) was used to measure segmental impedance values. To take a measurement, researchers entered the participant’s age, sex, and height into the TANITA machine, then requested participants to stand up straight on the weighing platform in bare feet (with feet in contact with the lower electrodes), while holding onto the hand grips (with hands in contact with the upper electrodes) until a complete impedance reading shows on the display. To reduce measurement variability, readings were taken immediately following a urinary void, and in the morning following an overnight fast. Within-day coefficients of variation for TANITA measures were <1%, while the coefficients of variation for measures repeated at 1 month (in a 5% repeatability subsample) were <5%. Height was measured twice to the nearest 1 mm using a portable stadiometer (Leicester height measure), circumferences (hip, waist, calf, head, chest inhaling, chest exhaling, mid arm) were measured twice to the nearest 1 mm using a non-stretch metallic tape measure, skinfolds (tricep, bicep, subscapular, suprailiac, calf) were measured three times to the nearest 0.2 mm using Holtain caliper, and grip strength was measured in kg four times using Lafayette 78010 hand-held dynamometer; the average of each of these measurements were used as a single value in analysis. DXA body composition values were measured from full-body scans using a Hologic Discovery A device with Hologic spine phantom 14855 used as a phantom. The participants were instructed to lay supine with their arms resting by their sides. Standard Hologic software defined the head, trunk, legs, and arms. DXA L1‐L4 regions were defined by marking the region from the midpoint of the T12 and L1 vertebrae to the midpoint of the L4 and L5 vertebrae in Hologic software [24]. The total mass, fat mass, and fat percentage were computed within this bounded region for each participant. Definition of the L1-L4 region was performed twice for each participant, and the average of these repeated values was used. This body segment has been used in previous studies as a more valid measure of abdominal fat than trunk fat, since the trunk includes additional body regions such as the chest and pelvis as well as the abdomen [25,26].

### Data preparation and statistical analysis

Participants were removed from the dataset if they had any missing data or if any of their measurement values were judged to be implausible suggesting machine or data entry error (see S1 File) for a list of the criteria applied). To account for potential bias/overfitting, the dataset was then split into training and testing groups by randomly selecting rows with 80% of individuals used to fit models and the other 20% used to validate the performance of the models. We explored use of the Least Absolute Selection and Shrinkage Operator (LASSO), random forest, and XGBoost algorithms for developing our prediction equations, as these represent popular parametric (LASSO) and non-parametric, tree-based (random forest, XGBoost) machine learning algorithms. The LASSO model provides the benefit of automatic feature selection, while random forest and XGBoost allow for more flexible modelling of non-linear relationships between predictors. The models were fit separately by sex, with the lambda value of the LASSO model, the mtry value of the random forest model, and the max depth, eta, and gamma parameters of the XGBoost model were tuned in the training data using 10-fold cross validation for each outcome. These algorithms were each used to fit models predicting 6 different DXA-derived outcomes: total body fat (kg), total body lean mass (kg), total body fat percentage (%), trunk fat percentage (%), L1-L4 fat percentage (%), and appendicular lean mass (kg). These six measures were selected as the focus of this paper as they are commonly reported in published research. For completeness, models to predict other measures estimated by the DXA machine (trunk fat mass (kg), trunk lean mass (kg), L1-L4 fat mass (kg), L1-L4 lean mass (kg), appendicular fat mass (kg), and appendicular fat mass percentage (%)) were computed in the same way.

The TANITA BC-418 device provides several outputs for the defined body segments of full body, trunk, left arm, right arm, left leg, and right leg. The following measurements as displayed in the TANITA output were used as predictors for each of these segments unless indicated otherwise: segment fat mass, segment fat-free mass, segment muscle mass (all but full body), segment fat percentage, segment impedance. Additionally, body weight, BMI, and total body water were used as predictors. Additional covariates included to potentially improve the predictive performance of the equations were age, standing height, calf circumference, head circumference, exhalation chest circumference, waist circumference, hip circumference, arm circumference, tricep skinfold, bicep skinfold, subscapular skinfold, suprailiac skinfold, calf skinfold, dominant hand grip strength, and non-dominant hand grip strength.

Certain data transformations and interactions were pre-specified for inclusion in the models if deemed to be relevant by the researchers based on review of the literature and potential utility in measuring muscle mass. These interactions and transformations were waist-to-hip ratio, waist-to-height ratio, chest-to-waist ratio, calf-to-height ratio, height-squared-to-impedance ratio for each impedance value, fat mass index (FMI), lean mass index (LMI), body shape index (BSI), corrected arm muscle area (CAMA), logarithm of the sum of skinfolds, and the product of age and dominant hand grip strength. Additionally, we originally included squared terms for each input variable to account for non-linear associations with the outcome and included additional speculative interaction terms to account for complex relationships between predictors. However, the inclusion of these terms did not result in considerable performance improvements, and in some cases worsened model performance due to overfitting. To maintain parsimony while still including potentially meaningful interactions, we used the reduced set of interaction terms described above in the present analysis.

Performance was compared in the 20% held-out testing data based on mean absolute error (MAE), mean average percentage error (MAPE), and root mean squared error (RMSE). RMSE was used to provide comparison to existing studies, while MAE and MAPE were utilized due to their interpretability. The built-in TANITA estimates for each outcome alone were used as a performance baseline. Since TANITA does not define the L1-L4 region, TANITA trunk values were used as the performance baseline for this outcome. Further, we compared the performance of our models to previously derived prediction equations by Kulkarni et al for use in Indian populations for the total body lean mass and appendicular lean mass outcomes [22] and to Durnin-Womersley equations for total body fat outcomes [20]. To provide further context for these evaluation metrics, we also estimated a ‘null’ or ‘baseline’ scenario by randomly permuting the DXA-based outcome values before splitting the data and evaluating model performance on these randomly permuted datasets (100 datasets were generated for total fat mass and 25 for the other outcomes due to computational burden).

As a post hoc sensitivity analysis, to understand which factors were contributing most to improved estimation of the new models, models were fit using the LASSO algorithm with only the full set of TANITA inputs and relevant transformations, full set of TANITA inputs along with one of the three sets of additional anthropometric variables and their transformations (skinfolds, circumferences, and grip strength), all circumferences alone, all circumferences and skinfolds and their transformations, and for comparison a full linear model containing all 63 covariates.

All analyses were completed using R software version 4.2.0.

## Results

**Fig 1:**
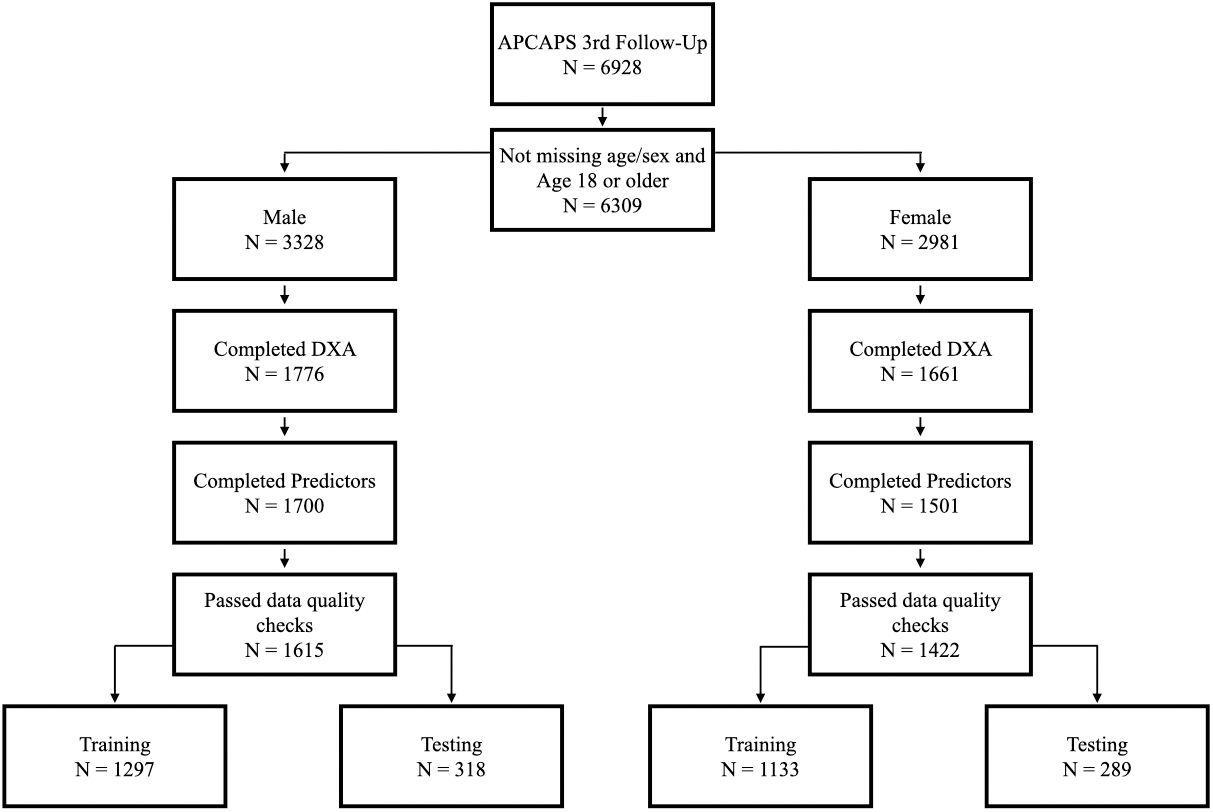
Flow-chart for inclusion in the present analysis.

The selection of study participants is described in a flowchart (Fig 1). The application of inclusion criteria resulted in a sample size of 1615 males and 1422 females (training dataset of 1297 males and 1133 females and validation dataset of 318 males and 289 females). Individuals who did not participate in the DXA study within APCAPS 3^rd^ follow-up tended to be younger on average than individuals who had DXA measurements available (34 years old and 38 years old, respectively), though BMI was similar in the two groups (mean 20.7 kg/m^2^ and 20.6 kg/m^2^, respectively). The characteristics of our final study cohort are described in Table 1. Males and females were of similar age (37 years and 38 years old on average, respectively, in both training and test datasets) and with similar BMI (approximately 20–21 kg/m^2^ in all groups). Within the same sex, the average value of each mass outcome (total body fat mass, total body lean mass, appendicular lean mass) did not differ by more than 0.51 kg between training and test datasets.

**Table 1:** Description of the sample.

	Training – Females (n = 1133)	Test – Females (n = 289)	Training – Males (n = 1297)	Test – Males (n = 318)
**Age (years)**	38.17 (11.7)	39.05 (11.3)	37.15 (15.5)	36.68 (15.5)
**Standing height (cm)**	151.68 (5.89)	151.20 (6.05)	164.26 (6.67)	164.27 (7.01)
**Weight (kg)**	48.57 (9.20)	48.39 (9.48)	55.46 (10.53)	54.46 (11.42)
**BMI (kg/m^2)**	21.14 (3.71)	21.22 (3.85)	20.55 (3.43)	20.15 (3.72)
**DXA Total body fat mass (kg)**	15.61 (5.21)	15.46 (5.25)	10.68 (4.98)	10.26 (5.17)
**DXA Total body lean mass (kg)**	32.03 (4.56)	32.00 (4.95)	43.52 (6.41)	43.01 (7.13)
**DXA Total body fat percentage (%)**	30.92 (5.55)	30.73 (5.72)	18.25 (5.64)	17.74 (5.67)
**DXA Trunk fat percentage (%)**	27.02 (6.98)	26.95 (7.16)	16.84 (6.56)	16.09 (6.52)
**DXA L1-L4 fat percentage (%)**	25.80 (7.71)	25.75 (7.92)	18.57 (7.69)	17.83 (7.64)
**DXA Appendicular lean mass (kg)**	13.11 (2.01)	13.01 (2.25)	19.18 (2.95)	19.04 (3.36)

All values presented as mean (SD)

The LASSO models achieved the best performance in the testing data for most of the 6 outcome-sex combinations (Table 2). Exceptions to this were for prediction of trunk fat percentage in males (MAE of 2.094% by LASSO vs MAE of 2.090% by XGBoost), L1-L4 fat percentage in males (MAE of 2.401% by LASSO vs MAE of 2.369% by random forest), total body fat percentage in females (MAE of 2.165% by LASSO vs MAE of 2.104% by random forest), and trunk fat percentage in females (MAE of 2.721% by LASSO vs MAE of 2.622% by random forest). Despite occasional performance gains by the random forest and XGBoost algorithms, we ultimately favor the LASSO algorithm for its comparative interpretability and ease of application to future data. We also noted that the LASSO algorithm performed similarly on the training compared with testing datasets (S2 File), whereas random forest and XGBoost algorithms performed markedly better on the training set across all outcomes, indicating overfitting, and further supporting robustness of the LASSO algorithm. Stratification of the test set by age (<40 vs 40 + years) indicated that the LASSO model performed well regardless of age, although performance was slightly superior in the younger group (S3 File).

**Table 2:** Mean Absolute Error (compared with DXA-based measurement) for different prediction algorithms using all predictors in test data, and additional performance metrics for best model (n = 289 female, 318 male).

Outcome (Sex)	Random Forest Mean absolute error	XGBoost Mean absolute error	LASSO Mean absolute error	LASSO Root Mean Squared Error	LASSO Mean Absolute Percentage Error
**Total body fat mass (kg) – M**	1.057	1.013	0.935	1.19	10.06%
**Total body fat mass (kg) - F**	1.014	1.052	0.976	1.26	6.94%
**Total body lean mass (kg) - M**	1.311	1.378	1.177	1.52	2.76%
**Total body lean mass (kg) - F**	1.308	1.357	1.222	1.55	3.85%
**Total body fat percentage (%) - M**	1.746	1.804	1.668	2.10	9.87%
**Total body fat percentage (%) - F**	2.104	2.125	2.165	2.73	7.44%
**Trunk fat percentage (%) - M**	2.121	2.090	2.094	2.62	13.81%
**Trunk fat percentage (%) - F**	2.622	2.652	2.721	3.42	11.15%
**L1-L4 fat percentage (%) - M**	2.369	2.417	2.401	2.99	14.92%
**L1-L4 fat percentage (%) - F**	2.971	2.970	2.929	3.75	12.41%
**Appendicular lean mass (kg) - M**	0.900	0.853	0.782	1.04	4.18%
**Appendicular lean mass (kg) - F**	0.833	0.860	0.790	1.00	6.17%

M is male; F is female.

Our novel equations, as well as the other equations previously developed by Kulkarni et al in an Indian population, performed substantially better than the built-in TANITA estimates and Durnin-Womersley equations (Table 3), and 3–4 times better than the ‘baseline’ scenario of randomly permuted datasets (S4 File). For 5 out of the 12 outcome-sex pairs, the mean absolute error of our best equation was less than half that of the TANITA built-in estimate or Durnin-Womersley equation. For example, mean absolute error for total body fat mass was 1.808 kg for males and 2.054 kg for females using TANITA estimation, 2.105 kg for males and 2.995 kg for females using Durnin-Womersley equations, 1.240 kg for males and 1.061 kg for females using our novel equation with just TANITA values, and 0.935 kg for males and 0.976 kg for females using our novel equation with TANITA, skinfolds, circumferences, and grip strength. The mean absolute error for a subset of the LASSO equations (TANITA predictors only, TANITA and skinfold as predictors, and full set of predictors), TANITA built-in estimates, and existing equations as compared to the DXA values are presented in Table 3. In all instances, the best performance in the test data was achieved by our novel equations (built using LASSO algorithm) including all measurements, that is TANITA, circumferences, skinfolds, and grip strength. These equations, which were based on between 14 and 31 selected features, performed similarly or better than a full regression equation based on all 63 features (S5 File). When exploring which features added the most value, we noted that similar performance was sometimes achieved through inclusion of only TANITA and skinfolds. The combination of TANITA and circumferences or TANITA and grip strength provided limited improvements upon the use of TANITA predictors alone.

**Table 3:** Mean Absolute Error (compared with DXA-based measurement) for the different prediction models in test data (n = 289 female, 318 male).

	Mean absolute error compared with DXA measurement				
Outcome (Sex)	TANITA Built-in estimate alone	Existing skinfold/ anthrop-ometric equation	All TANITA LASSO	All TANITA + Skinfolds LASSO	All TANITA + Grip strength + circumferences + skinfolds LASSO (I.e., Full LASSO)
**Total body fat mass (kg) - M**	1.808^a^	2.105^f^	1.240	1.003	0.935
**Total body fat mass (kg) - F**	2.054^a^	2.995^f^	1.061	1.016	0.976
**Total body lean mass (kg) - M**	2.782^b^	1.746^g^	1.401	1.230	1.177
**Total body lean mass (kg) - F**	2.670^b^	1.402^g^	1.276	1.234	1.222
**Total body fat percentage (%) - M**	3.242^c^	3.646^f^	2.202	1.801	1.668
**Total body fat percentage (%) - F**	4.256^c^	5.656^f^	2.298	2.193	2.165
**Trunk fat percentage (%) - M**	3.479^d^	---	2.574	2.298	2.094
**Trunk fat percentage (%) - F**	5.837^d^	---	2.922	2.823	2.721
**L1-L4 fat percentage (%) - M**	3.883^d^	---	2.859	2.596	2.401
**L1-L4 fat percentage (%) - F**	5.494^d^	---	3.184	3.081	2.929
**Appenciular lean mass (kg) - M**	1.778^e^	0.972^g^	0.926	0.826	0.782
**Appendicular lean mass (kg) - F**	1.548^e^	0.888^g^	0.811	0.804	0.790

M is male; F is female. a: TANITA fat mass; b: TANITA fat-free mass; c: TANITA total body fat percentage; d: TANITA trunk fat percentage; e: Sum of TANITA right arm fat-free mass, left arm fat-free mass, right leg fat-free mass, left leg fat-free mass; f: Durnin-Womersley skinfold equations; g: Kulkarni et al Indian-calibrated anthropometric equations.

**Fig 2:**
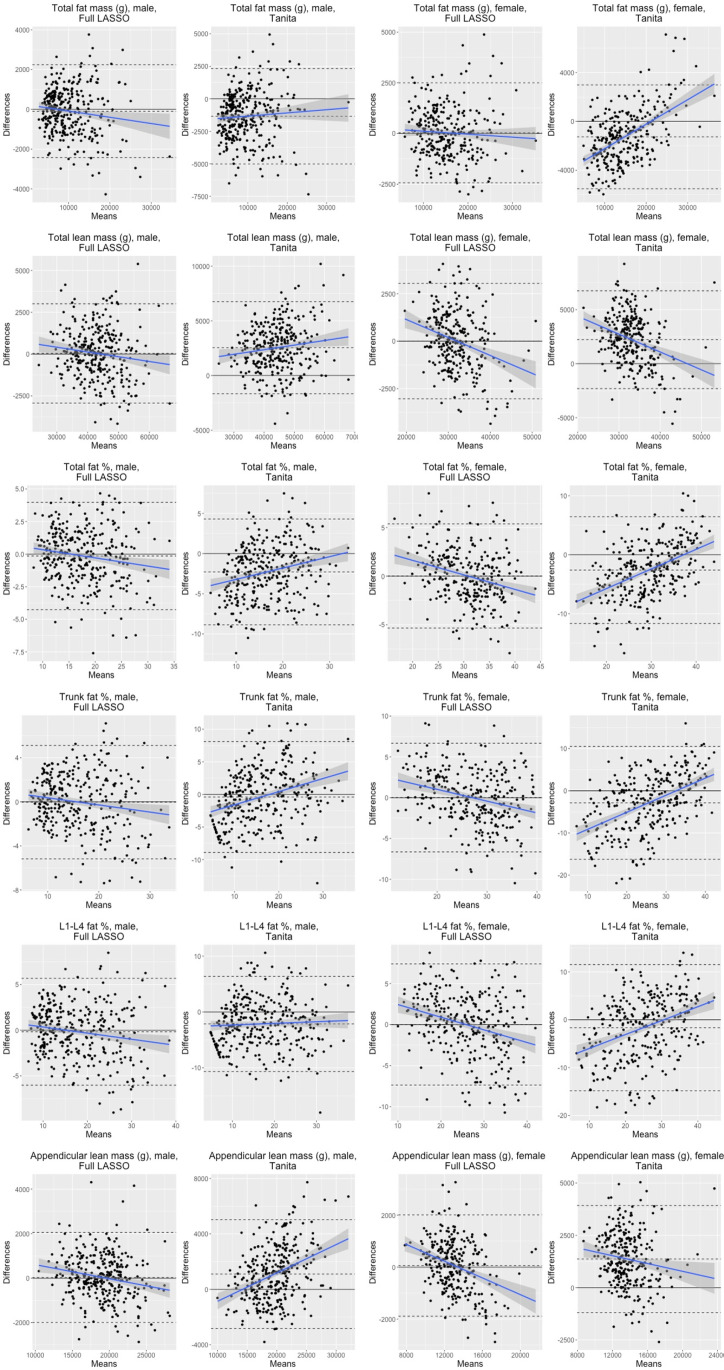
Bland-Altman Plots comparing the LASSO with all variables and TANITA Equivalent Measure alone to DXA values.

Bland-Altman plots were used to compare predictive performance of our full LASSO equations to that of the TANITA default outputs (Fig 2). Overall, there do not appear to be clear systematic errors in the predictions resulting from our equations, though total fat percentage in women and trunk fat percentage in both men and women may be frequently overestimated by our models among individuals with lower trunk fat percentage. Further, our equations may underestimate appendicular lean mass among women with higher appendicular lean mass. The TANITA device appears to frequently underpredict the fat mass of women with lower body fat mass.

The coefficients for each outcome-sex pair in the LASSO model with all sets of predictors are provided in the S6 File. Performance metrics for additional body composition metrics (e.g., appendicular fat mass) which were not the focus of this manuscript are provided in S7 File. The 1^st^ and 99^th^ percentiles of each predictor in the training data are provided in S8 File. All LASSO equations can be used through an interactive application designed by the authors and hosted on GitHub (see data sharing statement).

## Discussion

We have developed and validated the performance of a novel set of equations to predict six different clinically important DXA-derived body composition metrics in Indian adults from low-cost BIA plus anthropometric measures. In all cases our novel equations performed substantially better than BIA estimates or anthropometric equations developed in other ethnic groups, reducing mean errors by over 50%. Of the anthropometric measures added (circumferences, grip strength and skinfold thicknesses), skinfold thicknesses made the largest contribution to improved estimation, especially among males. This demonstrates that although BIA measures may be inaccurate for Asian Indian populations, they can be recalibrated successfully with the addition of simple anthropometric measures. Our equations will be useful for improving body composition estimation in large-scale studies in India, and our approach could be adapted for re-calibration of other BIA devices and for other under-represented ethnic groups.

While we identified several studies validating existing body composition metrics for use in Indian populations, our study represents one of only a few studies developing novel equations to predict DXA-derived values for use with Indian adults. While Dasgupta et al provided similar equations with lower reported RMSE than our study, their study sample consisted only of 117 males aged 18–22 years old [19]. As such, the generalizability of these equations may be more limited than the novel equations presented in the current work, which we developed using a training sample of more than 1000 men and women of a wide age range, the largest population-based DXA dataset including both men and women available in India to our knowledge. Grover et al developed equations to predict DXA-derived fat mass and fat-free mass using BIA, circumferences, and skinfolds separately in a clinical population of 302 adults with cirrhosis but did not present equations using a combination of all factors [27]. Equations previously developed by our group for lean mass used skinfolds and waist circumference but did not explore the addition of BIA values into predictive equations [22]. Although the performance for appendicular lean mass was within 0.1 kg of our equations using TANITA only, this is somewhat expected as they used data from some of the same individuals. Prior development of equations for fat-free mass based on a sample of Black and White individuals by Sun et al reported RMSE of 3.9 kg for males and 2.9 kg for females [6], while the equations developed by Rush et al for estimating fat-free mass in an Asian Indian population report an RMSE of 2.13 in the study sample [18]. The RMSE for our LASSO-based equations predicting total lean mass were 1.5 kg for males and 1.6 kg for females, indicating superior performance than previous attempts. To our knowledge, the current work represents the introduction of the first set of validated BIA-based equations to predict trunk-specific body composition outcomes for Indian adult men and women, which is timely considering concern around increased central adiposity in this population. Interestingly, our equations calibrated for this Indian population performed better than the device-generated BIA measures perform against DXA in recent validation studies in white and east Asian populations (in whom the devices’ in-built equations were developed) [28–30], raising the possibility that skinfolds and other anthropometric measures could be useful in improving body composition estimation from BIA in all contexts.

Though we have demonstrated improved performance from the TANITA output in estimating lean mass in both the full body and appendicular regions, it is unsurprising that the TANITA outputs have a noticeable systematic error. This is because the TANITA uses a two-compartment model to predict fat-free mass, combining both lean mass and skeletal mass into a single measure, whereas the DXA-derived outcome specifically uses a three-compartment model to measure lean mass as distinct from skeletal mass. Additionally, the TANITA provides no direct measure of L1-L4 fat mass as the device cannot detect this body segment, while the TANITA trunk segment includes the mass of the head, while the DXA includes the head as a separate body segment. These observed systematic errors may explain the performance issues of the TANITA observed in our study, though they also emphasize the relevance of the current work. Those interested in measuring lean mass without skeletal mass using TANITA now have a method of doing so despite its use of a two-compartment model.

Our work is not without limitations. Not all participants from APCAPS attended the clinic for DXA measurements due to funding limitations or travel distance to the clinic. The participants who did not attend DXA clinics tended to be younger than the participants who did attend the clinics, suggesting there could be systematic differences between those included and those not included due to missing measurements, though no significant difference was found in BMI between the individuals with and without DXA measurements. These equations are not recommended for estimating body composition among pregnant women since these women were not included in the sample used for the present development and validation of equations. The validity of these equations in the presence of values outside the range of the training data is also unclear. The training dataset mostly consisted of low-BMI individuals (99^th^ percentile for BMI in both men and women ≈ 31 kg/m^2^). Further, the 99^th^ percentile for age in our data is 67 years in men and 61 years in women. Therefore, predictions may not be suitable for adults with obesity as defined by BMI > 30 kg/m^2^ and those older than the study sample, and we recommend interpreting model predictions with caution in such cases.

Beyond the limitations related to the distribution of variables and selection into the DXA study, limitations to interpretation of results also arise from the use of an internal, rather than external, validation dataset. Our participants are largely from peri-urban areas in southeastern India, and it is unclear how well these equations will perform in other populations in India and in the diaspora, as India is an ethnically and culturally diverse country. Additionally, most participants in the APCAPS cohort were between 18–30 or 40–55 years old, meaning that individuals 30–40 years old were underrepresented in the dataset. Because of these limitations, future work is needed to validate these equations in other Indian cohorts and among adults of Indian ethnicity living outside of India. Further, while prior research has shown that DXA is a good substitute for MRI-derived measures of abdominal fat in Indian populations, some researchers still favor use of MRI as a gold standard for abdominal fat, since DXA may tend to overestimate fat mass in leaner individuals [24].

Finally, future validation work is crucial to assess the validity of these equations when deriving BIA estimates from other devices. The present study made use of the TANITA BC-418 device, which is no longer manufactured having been replaced by newer models such as the MC-780U (although older devices such as the BC-418 are still in circulation, especially in low-resource settings where new devices may be less affordable). This newer model has been described by the manufacturer as a direct replacement and provides the same 50KHz measurement frequency, suggesting our equations could be used across both devices. Further, the additional measures provided by the new model, such as measurement of impedance using additional frequency values, may present opportunities for development of equations using an expanded set of inputs in the future. Additionally, validation work is needed to assess the performance of these equations when using BIA-based inputs from manufacturers other than TANITA in case of systematic differences between devices. It is the authors’ expectation that the equations will perform well with inputs from any BIA device that can provide segmental estimates, as the underlying approach to impedance estimation is similar, and studies have reported high correspondence in body composition estimates across manufacturers, although some studies have suggested estimates from Omron devices and InBody BIA devices may differ from other manufacturers [31,32]. There are many commercially available BIA device manufacturers for which updated models are frequently released; our extensive data-driven analyses of a large dataset provide an approach which could be used to improve calibration of existing and new devices. We believe that BIA device manufacturers will be very interested in these findings as a means to improve the accuracy of their devices for populations with distinct body phenotypes. We note that our equations are not suitable for use with BIA devices which provide only full-body measurements, as information about the separate body segments are required as input.

## Conclusions

In summary, we have used machine learning-based approaches to develop equations capable of markedly improving body composition estimation for Indian adults. The inputs to our equations are measures from a commercial BIA device plus simple anthropometric measurements, and thus have potential to be used in large-scale research studies, or in primary/community care settings where advanced body scanning devices are not available. This serves as a proof of principal for other populations for whom in-built equations in BIA devices perform poorly (such as other non-white ethnic groups). We have made our equations openly available so that they can be used by the wider research community and contribute to improved understanding of the role of body composition in disease risk in the Indian population [33].

## Supporting information

S1 File List of data quality rules applied for inclusion in the study. (DOCX)

S2 File Performance metrics (compared with DXA-based measurement) for different prediction algorithms using all predictors in training data. (CSV)

S3 File Performance metrics (compared with DXA-based measurement) for different prediction algorithms using all predictors in test data, overall and stratified by age (<40 years test n = 122 female and 185 male, 40 + years test n = 167 female and 133 male). (CSV)

S4 File Performance metrics (compared with DXA-based measurement) for different prediction algorithms using all predictors in test data, based on 25 datasets where the DXA-based outcomes were randomly permuted (to provide a null or baseline scenario to compare against performance on the real data). (CSV)

S5 File Performance (Mean Absolute Error) of the LASSO with alternate sets of predictors. (CSV)

S6 File Coefficients for each outcome in full model. (CSV)

S7 File Information about model performance for other outcomes (trunk fat mass (kg), trunk lean mass (kg), L1-L4 fat mass (kg), L1-L4 lean mass (kg), appendicular fat mass (kg), and appendicular fat mass percentage (%)). (CSV)

S8 File Minimum, 1st percentile, 99th percentile, and maximum of each predictor in the training data. (CSV)
