# Supplementary material for: Machine learning-based equations for improved body composition estimation in Indian adults
Source: PLOS Digit Health. 2025 Jun 23;4(6):e0000671. doi: 10.1371/journal.pdig.0000671 (PMC12185013; doi:10.1371/journal.pdig.0000671)
Supplement: S1 File — (DOCX) [file pdig.0000671.s001.docx]

S1 File: List of data quality rules applied for inclusion in the study

- Head circumference cannot be less than 45 cm
- grip strength cannot be more than 100 kg
- body fat percentage as measured by either device cannot be larger than 50%
- Tanita total weight must be within 1 kg of the sum of fat mass and fat-free mass
- Tanita muscle values for each body segment cannot be larger than fat-free mass
- Tanita total fat mass and fat free mass must be within 1 kg of the sum of each body component
- Fat mass must be within 2kg of component sum for DXA, lean mass must be within 5kg of component sum for DXA, and total mass must be within 7kg of component sum for DXA since head is not included as a body segment in our data
- Body cannot be less than 10% water in Tanita
- Muscle mass of right and left arm or leg cannot differ by more than 5 kg
- Impedance cannot be less than 100 Ohms for whole body or greater than 1000 Ohms for any limb
- Difference between weight from scale and Tanita or DXA must be less than 3kg.
